# Real-time remote outpatient consultations in secondary and tertiary care: A systematic review of inequalities in invitation and uptake

**DOI:** 10.1371/journal.pone.0269435

**Published:** 2022-06-03

**Authors:** Janet E. Jones, Sarah L. Damery, Katherine Phillips, Ameeta Retzer, Pamela Nayyar, Kate Jolly

**Affiliations:** 1 Institute of Applied Health Research, University of Birmingham, Birmingham, United Kingdom; 2 Centre for Patient Reported Outcomes Research, Institute of Applied Health Research, University of Birmingham, Birmingham, United Kingdom; Syddansk Universitet, DENMARK

## Abstract

**Background:**

Health policies in most high income countries increasingly recommend provision of routine outpatient care via remote (video and/or telephone) appointments, especially due to the pandemic. This is thought to improve access to care and promote efficiency within resource-constrained health services. There is limited evidence about the impact on existing inequalities in the invitation and uptake of health services when remote outpatient care is offered.

**Aim:**

To systematically review the evidence on the offer and/or uptake of real-time remote outpatient consultations in secondary and tertiary care, assessed according to key sociodemographic characteristics.

**Methods:**

Seven electronic bibliographic databases were searched for studies reporting the proportion of patients with key characteristics (following PROGRESS Plus criteria) who were offered and/or accepted real-time remote outpatient consultation for any chronic condition. Comparison groups included usual care (face-to-face), another intervention, or offer/uptake within a comparable time period. Study processes were undertaken in duplicate. Data are reported narratively.

**Results:**

Twenty-nine studies were included. Uptake of video consultations ranged from 5% to 78% and telephone consultations from 12% to 78%. Patients aged over 65, with lower educational attainment, on lower household incomes and without English as a first language were least likely to have a remote consultation. Females were generally more likely to have remote consultations than males. Non-white ethnicities were less likely to use remote consultations but where they did, were significantly more likely to choose telephone over video appointments (p<0.001).

**Conclusions:**

Offering remote consultations may perpetuate or exacerbate existing health inequalities in access to healthcare. More research is needed on current health disparities by sociodemographic characteristics and to explore what works well for different patient groups and why so that processes can be designed to ameliorate these health disparities.

**Trial registration:**

PROSPERO registration no: CRD42021241791.

## Background

The established model of face-to-face outpatient care in the hospital setting has remained largely unchanged in the UK since the inception of the National Health Service (NHS). Data suggest that outpatient care costs the NHS over £8 billion a year, and an upward trend in demand is set to increase substantially as the population ages and the incidence of chronic conditions rises [1]. In response to these pressures and to improve patient access to care and enhance clinical efficiency the 2019 NHS long-term plan aims to reduce face-to-face outpatient appointments by a third by 2024 [2]. The offer of a remote consultation enables convenient access to healthcare for patients by removing barriers such as having to travel, sometimes quite long distances, to attend a face-to-face consultation and having to take time off work. Recently, the need to continue to deliver healthcare to those requiring it despite Government restrictions on person to person contact during the COVID-19 pandemic [3] meant that health services rapidly had to adapt the manner in which outpatient care was delivered. Consequently, remote consultations, in which patient-clinician interactions took place over the telephone or a video link, were swiftly implemented for the majority of outpatient appointments both in the UK and in other developed economies [4, 5].

During 2020, the use of digital technologies for hospital outpatient appointments in the UK rose from 2019 levels of approximately 200 per day, to over 6000 per day [3]. Whilst remote consultations are argued to offer improved access to care, convenience, savings on patient travel and time off work costs [6, 7], greater clinical efficiency [8], and high patient satisfaction [7, 9], it is important that the rapid and widespread adoption of remote consultations does not widen or exacerbate existing health inequalities and that the offer and uptake of remote consultations is equitable. For example, it has been argued that remote consultations may disadvantage those with poor technology access [10]. Although 96% of households in the United Kingdom (UK) had internet access in 2020, this drops to 80% for households with at least one occupant aged 65 or over [11]. Furthermore, many individuals may lack the digital skills required to enable them to safely and confidently navigate the digital world [12, 13] or poor internet infrastructure may prevent them from having real-time consultations [14, 15]. Patients from disadvantaged backgrounds and those not speaking English as a first language tend to be disenfranchised from remote consultations [16, 17].

A number of studies have evaluated the effectiveness of remote consultations in primary care [10, 18, 19], including a recently published systematic review of health inequalities relating to remote appointments in this setting [4]. Although studies evaluating remote consultations in secondary care or tertiary care exist [6, 16, 20], there is a gap in the literature assessing the extent to which remote consultations in these settings may exacerbate or alleviate inequalities in offer and uptake of remote outpatient healthcare. The aim of this study was to carry out a systematic review of observational or intervention studies reporting data on the proportion of patients a) invited to, and b) participating in remote outpatient consultations in secondary and tertiary care, and to assess whether these differ according to sociodemographic characteristics known to affect health inequalities.

## Methods

The findings are reported following the PRISMA guidelines for the reporting of systematic reviews [21]. Ethical approval was not required. A protocol was registered with PROSPERO, registration no: CRD42021241791.

### Definitions

‘Remote consultation’ refers to any real-time (synchronous) consultation between patient and clinician that takes place at a distance rather than in-person and face-to-face. These can be carried out over the telephone or via video technology. ‘Invitation’ or ‘offer’ refers to any communication received by patients from healthcare providers asking them to attend a remote consultation. ‘Uptake’ refers to whether patients take up the option to have a remote consultation when offered. Chronic illness, for the purposes of this review, is defined as a health condition lasting for a long period and which can worsen over time. Examples include: diabetes, asthma, arthritis and COPD [22].

The definition of ‘health inequalities’ includes those defined by the PROGRESS-PLUS criteria [23, 24] and/or NHS England’s definitions of health inequalities [25], such as homeless individuals, rough sleepers, refugees, asylum seekers and those from traveller communities. The World Health Organisation (WHO) defines health inequality as “differences in health status or in the distribution of health determinants between different population groups” (e.g. racial, ethnic, age group, gender, socio-economic group, sexual orientation) [26]. National and regional social and economic conditions can impact on the social determinants of health resulting in a disparity in the risk of illness and/or the treatments provided for different sectors of society [27]. Many people will experience more than one inequality (e.g. older, disabled, minority group, sexual orientation, religion) which could compound the extent to which they may face challenges in accessing health services [24]. To illustrate the multifaceted aspects of health inequalities Evans and Brown developed the PROGRESS criteria which were later expanded into the ‘PROGRESS-PLUS’ criteria. [23]. The additional PLUS criteria include personal characteristics associated with discrimination (e.g. age, disability), features of relationship (e.g. smoking parents, exclusion from school) and time-dependent relationships (e.g. leaving hospital, respite care, and other instances where a person may be temporarily at a disadvantage).

### Study designs and settings

The review included any observational or interventional study designs reporting data on the proportion of patients with key sociodemographic characteristics who were offered and/or accepted remote consultation(s) for their outpatient care. The conditions of interest covered any chronic illness. Mixed methods studies were included if quantitative data could be extracted; studies reporting solely qualitative or narrative data were excluded. Studies undertaken in low and middle income countries (LMIC) were also excluded (identified using the Cochrane Collaboration LMIC filter: https://datahelpdesk.worldbank.org/knowledgebase/articles/906519-world-bank-country-and-lending-groups), as the findings from studies in LMIC may have limited applicability outside of those settings. Studies that included primary care data were still eligible if standalone secondary and/or tertiary care data could be extracted. Table 1 outlines the review inclusion and exclusion criteria.

**Table 1:** Inclusion and exclusion criteria.

**Inclusion criteria**
Adult outpatients in secondary care
Adult outpatients in tertiary care
Invitation to/offer of a synchronous remote consultation
Telephone consultations
Video consultations
High income countries
Published in English
Published since 2010
Any chronic illness
Observational or interventional study designs
Mixed-methods studies (if they included extractable quantitative data)
Studies reporting on any of the following characteristics: Age, gender, ethnicity, income, educational attainment, employment status, social economic status, first language and area of residence (rural or urban).
**Exclusion criteria**
Studies focusing on mobile health (mHealth) interventions
Papers describing IT and/or software infrastructure
Video technology used during surgery or as part of healthcare professional teaching/training
Remote consultations used purely for diagnostic purposes rather than patient follow-up
Group interventions (e.g. remotely delivered weight management groups)
Hypothetical studies (e.g. surveys asking patients whether they would accept a remote consultation if offered)

### Population

Adult patients receiving routine outpatient care for any chronic condition through secondary or tertiary care, who were invited to and/or participated in a remote consultation.

### Intervention

Real-time outpatient appointments between a patient and one or more healthcare professionals within or across secondary and tertiary care settings using video technology or telephone.

### Comparator

Data for one or more comparison groups were not essential for study inclusion. Relevant comparison groups, where reported, were usual care (face to face, in-person appointments), comparison to another intervention, or offer/uptake of remote consultations during a comparable time period (e.g. specific months in one year compared with the same months in a subsequent year).

### Outcomes

The outcomes of interest were:

1. The proportion of people invited to attend and/or participating in a remote consultation
2. The proportion of people participating in remote consultations by appointment type (i.e. video or telephone)
3. The proportion of people requiring a follow-up in-person consultation after a remote consultation
4. The rate of non-attendance of a remote consultation

### Searches

Seven electronic bibliographic databases were searched: Medline, Embase, PsycINFO, Health Management Information Consortium (HMIC), Applied Social Sciences Index and Abstracts (ASSIA), CINAHL, Social Science Citation index. The search strategy included general and medical subject headings (MeSH terms) related to remote consultations (e.g. teleconsultations, virtual consultations, e-consultations), and socioeconomic status, with search terms modified appropriately for each database (S1 File). Searches were undertaken in February 2021 (updated November 2021), and limited to papers published in the English language and after the year 2010 as scoping searches indicated the use of remote consultations was uncommon before this date.

### Study selection and screening

Search results were transferred to the Rayyan QCRI central electronic reference management application (https://www.rayyan.ai) and duplicates removed [28]. Titles and abstracts were split into equal batches and each batch was independently screened for relevance by a pair of reviewers from a pool of five (JJ, SD, KT, AR, PN). Disagreements were resolved by discussion. Full text screening for all potentially eligible studies was independently undertaken by two reviewers (JJ, SD). Disagreements were again resolved by discussion and consultation with the wider research team if uncertainty over eligibility remained.

### Data extraction

One reviewer (JJ) completed data extraction for all papers using a pre-developed data extraction form (S2 File). Data were extracted on study characteristics (design, data source(s), dates of data collection, setting, disease area), intervention and comparator, sociodemographic characteristics reported (age, ethnic group, gender, socioeconomic status, area of residence (urban/rural), language, education, household income, employment status), and data on each of the relevant outcomes. Two further reviewers (SD and PN) independently checked all data extractions to ensure consistency and accuracy in the data extracted.

### Quality assessment

As studies with either randomised or observational study designs were eligible, the methodological quality of included studies was appraised using the Mixed-Methods Appraisal Tool (MMAT) (S3 File) which allows for critical quality appraisal of both quantitative and mixed methods studies, covering study design, sampling, selection biases, between-group comparisons, measurements and response rates [29, 30].

### Data synthesis and analysis

After testing for statistical heterogeneity, the authors concluded that meta-analysis of study data was inappropriate. Therefore, formal statistical analysis was not carried out. All data in this manuscript (e.g. proportions of individuals overall and by clinical/sociodemographic sub-group within a study who participated in remote consultations, and analyses to assess statistically significant similarities and/or differences between groups) are those reported by the authors of the included studies. Data are reported narratively. For the purposes of clarity the authors’ original terminology were preserved (S6 File).

### Patient and Public Involvement (PPI)

The patient and public group of the National Institute for Health Research (NIHR) Applied Research Collaborations (ARC) West Midlands Long-term Conditions Theme were consulted about the review and provided input to the protocol, formulation of the research questions, and advised on which outcomes may be considered the most important to patients. The group also provided input into the interpretation of the review findings.

## Results

### Study selection

**Fig 1:**
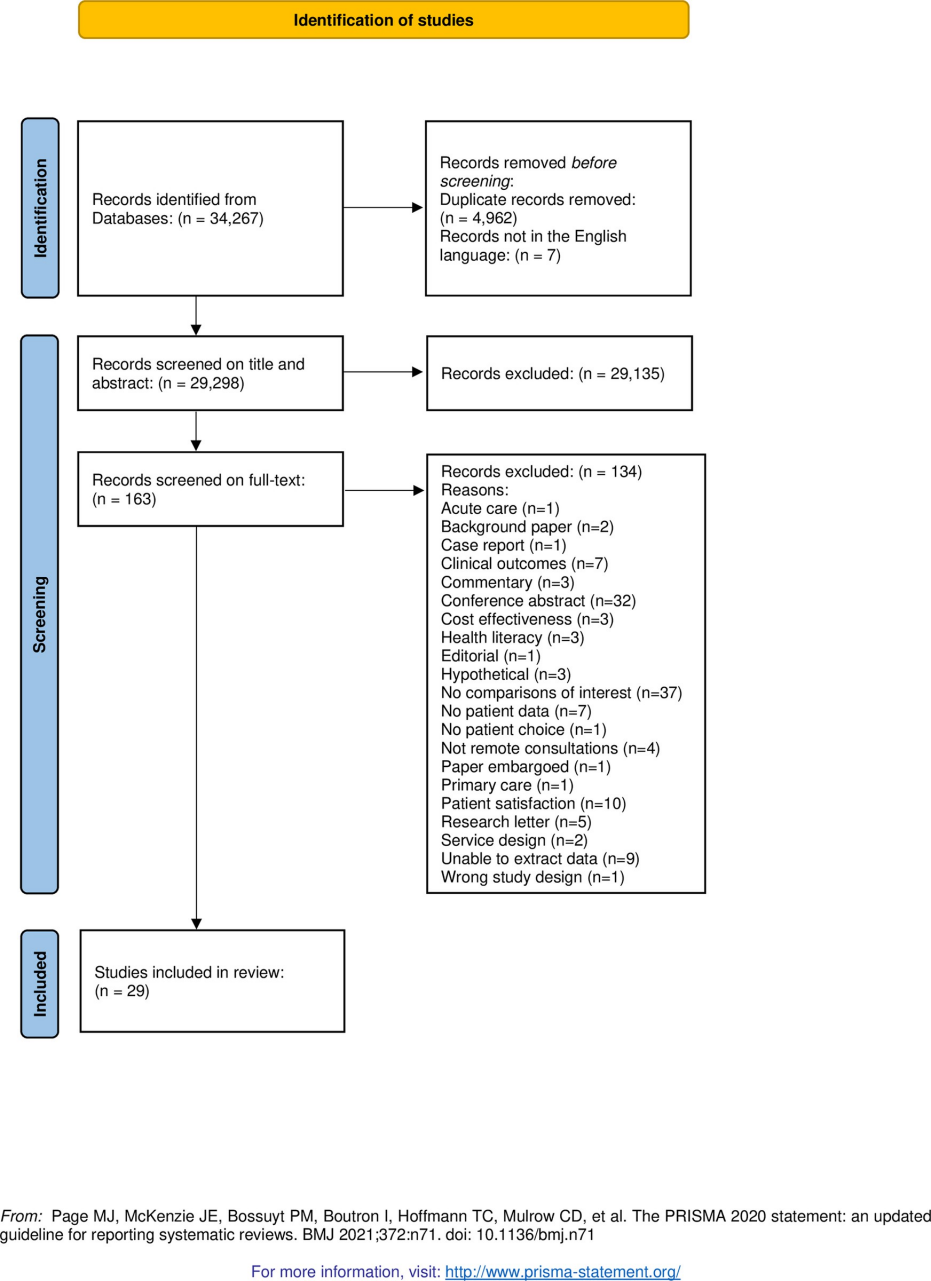
Study selection process.

Searches of the bibliographic databases returned 34,267 possible studies. There were 4,962 duplicates and seven not in the English language. Title and abstract screening was carried out on the remaining 29,298 studies, from which 163 citations were taken forward for full-text screening. Twenty-nine studies were included in the review (Fig 1). Of the 29 included studies, 21 were identified in the initial phase of the review; eight were identified during the review update. (S4 File, PRISMA checklist).

### Quality of included studies

All 29 studies were judged to be of moderate to high quality (S5 File). In some cases not all data were available; there were difficulties separating some of the reported data into different appointment types, or the analytical approach was not described.

### Description of included studies

All 29 studies were published between 2017 and 2021 with the majority (n = 27) published within the last two years. Twenty-one studies were based in the USA [31–51], three in Australia [52–54], two in Canada [55, 56], and one each in Chile [57], Italy [58] and Scotland [59]. One based their research in primary and secondary care settings [37], one in primary and tertiary care [44], eleven solely in secondary care [39, 41, 45, 46, 51, 52, 55–59] and sixteen in tertiary care [31–36, 38, 40, 42, 43, 47–50, 53, 54]. The research was specifically set in the context of COVID-19 in twenty-one studies [32, 33, 35–39, 41, 43–51, 54, 56–58]. Four studies reported on the number of participants offered a remote consultation [32, 42, 43, 52], two reported the number of patients who declined a remote consultation [42, 43] and three assessed the number of patients who failed to attend their appointment [40, 52, 59]. Only one study reported separate recruitment numbers for new and established patients [32]. The remaining studies only reported data on those who attended an appointment. There was heterogeneity in medical conditions covered. Twenty-two studies considered a single clinical specialty; the remaining seven included patients with any condition or those with chronic conditions in general. A retrospective cohort study was the most frequent study design [31, 36–41, 44, 45, 47, 49, 55]. All but one study reported data on participants’ gender [49], with twenty reporting ethnicity data [31–33, 35–42, 44–46, 48–51, 53, 55] and fifteen mean age [31–35, 39, 42, 46, 48, 51–54, 57, 58] (Table 2). Twenty one studies reported data from a single time period [31–34, 36, 37, 39, 40, 42–44, 47, 49, 52–59], and eight reported a comparison of remote consultation uptake rates across two time periods [35, 38, 41, 45, 46, 48, 50, 51].

**Table 2:** Summary of included studies.

				Reported participant characteristics									
Author (Year)	Condition(s)	Study Design	Data source	Age	Age group	Gender	Ethnicity	Income	Education	Employment	SES	Language	Urban/rural
Abel (2018)	Mental health	RC	D/b	Mean	Y	Y	Y	N	N	N	Y	N	Y
Adeli (2021)	Opthalmology	CS	EHR	Mean	N	Y	Y	N	N	N	N	Y	N
Almandoz (2021)	Obesity	RV	CR	Mean	Y	Y	Y	Y	Y	N	N	N	N
Andino (2020)	Urology	MC	EHR	Mean	N	Y	N	N	N	N	N	N	N
Chunara (2021)	COVID-19	C	EHR	Mean	N	Y	Y	N	N	N	N	Y	N
Darrat (2021)	Otolaryngology	RC	EHR	Median	N	Y	Y	N	N	N	N	N	N
Eberly (2020)	Chronic illness*	RC	EHR	N	Y	Y	Y	N	N	N	N	Y	N
Franciosi (2021)	Chronic illness	CS	EHR	Mean	N	Y	Y	N	N	N	N	Y	N
Gilson (2020)	Any	RC	EHR	N	Y	Y	Y	N	N	N	N	N	N
Irarrazavel (2020)	GI surgery	P	D/b	Mean	N	Y	N	N	N	N	N	N	N
Jaffe (2020)	Any	RC	IC	Mean	Y	Y	Y	Y	Y	Y	N	N	Y
Kemp (2020)	Abdominal surgery	RC	EHR	N	Y	Y	Y	N	N	N	N	N	N
Lepage (2020)	Hepatitis C	RC	EHR	N	N	Y	Y	N	Y	N	Y	N	Y
Lewis (2021)	Neurology	F	S	Mean	N	Y	N	N	N	N	N	N	N
Liu (2021)	Geriatric medicine	CS	EHR	Median	N	Y	N	N	Y	N	N	Y	N
Lonergan (2020)	Cancer	RC	EHR	Median	N	Y	Y	N	N	N	N	N	Y
Menon (2017)	Diabetes	CS	S	Mean	N	Y	Y	N	N	N	N	N	N
Moo (2020)	Dementia	R	S	Mean	N	Y	Y	N	N	N	N	N	N
Ohlstein (2020)	Otolaryngology	P	D/b	Median	N	Y	N	N	N	N	N	N	N
Poeran (2021)	Chronic illness	RC	IC	N	Y	Y	N	Y	N	N	N	N	Y
Rodgriguez (2021)	Chronic illness	RC	IC	N	Y	Y	Y	Y	Y	N	Y	Y	N
Rowe (2021)	Cardiology	CS	EHR	Mean	N	Y	N	N	N	N	N	Y	Y
Santonicola (2020)	Liver	P	D/b	Mean	N	Y	N	N	Y	N	N	N	N
Sellars (2020)	Colorectal	P	D/b	Median	Y	Y	N	N	N	N	N	N	N
Shehan (2021)	Otolaryngology	R	EHR	Mean	N	Y	Y	Y	N	N	N	Y	N
Stevens (2021)	Chronic illness	RC	EHR	N	Y	N	Y	N	N	N	N	N	N
Wegerman (2021)	Liver	RC	D/b	Median	N	Y	Y	N	N	N	N	N	N
Xiong (2021)	Orthopaedics	R	EHR	Median	N	Y	Y	Y	N	N	N	Y	N
Yuan (2021)	Cardiology	CS	EHR	Mean	N	Y	Y	N	N	N	N	N	N
**Totals**				**22**	**10**	**28**	**20**	**6**	**6**	**1**	**3**	**9**	**6**

Key: C = cohort, CR = chart review, CS = cross-sectional, D/b = database, EHR = electronic health records, F = feasibility, IC = insurance claims, MC = matched cohort, N = not included, P = prospective, R = retrospective, RC = retrospective cohort, RV = retrospective review, S = survey, SES = socio-economic status, Y = included.

### Uptake of remote consultations

Twenty-eight studies reported uptake of video consultations, with uptake rates ranging from 5% [31] to 78% [40]. Eight studies reported on the uptake of telephone consultations ranging from 12% [36] to 78%

### Uptake of remote consultations pre- vs. post-COVID-19

Twenty-five studies provided data assessing changes in remote consultation uptake over time. Seven studies reported pre-COVID-19 data only [31, 40, 42, 47, 53, 55, 59], two compared pre- and post-COVID-19 time periods [35, 41], and 16 reported data during the post-COVID-19 period [32, 33, 36–39, 43–45, 49–51, 54, 56–58]. All but one of the pre-COVID studies reported uptake rates less than 20%. Conversely, Kemp reported an uptake rate of 78% for remote outpatient consultations following abdominal surgery [40]. The two studies reporting pre- and post-COVID-19 data each showed an increase over time, from 0% to 27% in one study specifically focused on COVID-19 [35], and from 10% to 63% in the other [41] which reported oncology data over time. In the 16 studies reporting post-COVID-19 data only, rates of remote consultation ranged from 6% [39] to 84% [50] with all but two of the sixteen studies reporting an uptake to remote consultation of >20%.

### Patient socio-demographics and remote consultations

S6 File summarises the characteristics and findings of the included studies.

#### Age

All but one study [55] reported the overall mean/median age of patients or reported age categories. Seventeen studies significantly associated older age with reduced use of remote consultations [31, 33, 34, 36–39, 42–47, 49, 51, 54, 58], with five of these reporting that older patients were less likely to participate in video appointments compared to telephone appointments [36, 38, 44, 49, 51]. For example Yuan states that those seen by video consultation had a significantly younger mean age compared to those having telephone consultations (p<0.001). Conversely, four studies reported no difference in age between the uptake of telehealth and usual care appointments [52, 56, 57, 59].

#### Gender

Patient gender was reported in 28 studies, only the study by Stevens did not report this characteristic [49]. In general, females were significantly more likely to attend telehealth visits compared to males, however, five studies found no difference in remote consultation attendance by gender [45, 47, 50, 56, 59]. For example, Xiong reported no difference in gender between the patients using remote consultations (p = 0.66), whereas Andino reported females to be significantly more likely to have a video consultation than males (p = 0.0013). One study reported that more males attended remote consultation appointments but there was no significant difference between genders for usual care visits [42]. Eberly et al. found that females were more likely to attend primary care remote consultations than those in secondary or tertiary care and that these were significantly more likely to be telephone rather than video consultations (p<0.001, OR 0.92 [95% CI, 0.90–0.95]), [37].

#### Socioeconomic status

Eight studies reported that participants who were insured, employed, had higher household income, and a higher educational attainment were more likely to have a remote consultation compared to those with lower household incomes, lower education or who were uninsured [31, 33, 39, 44, 47, 50, 56, 58]. Conversely, two studies reported that those on a low income and/or who were uninsured were accepting of technology [32, 48]; the difficulty for one study was contacting patients in these groups to offer a remote consultation [32]. Similarly, Lepage observed that those patients using remote consultations were more likely not to have graduated high school compared to those receiving usual care or mixed method delivery (17% versus 11% versus 14%) (p<0.0001) [55]. Poor access to technology, broadband, suitable devices and lower digital literacy were highlighted as reasons for lower engagement with remote consultations [38, 42, 44].

#### Ethnicity

Data on ethnicity were reported in twenty studies [31–33, 35–42, 44–46, 48–51, 53, 55]. Of these one study was based in Australia [53], one in Canada [55], and the remainder the USA. Although in each of these studies, patients with white ethnic backgrounds formed the ethnic majority, African American, black, Hispanic, Latino and Asian patients were significantly less likely to have a remote consultation or more likely to fail to attend or complete a remote consultation, [31, 37, 40, 44–46, 49, 50] as were those whose first language was not English. [37, 44, 50] Two studies observed that remote consultations served a greater proportion of Indigenous patients compared to other ethnicities; for instance Menon reported that there were a higher proportion of indigenous patients in the telemedicine group compared to the in-person group (p<0.001) [53, 55]. A significant increase in the uptake of remote consultations during COVID-19 compared to pre-COVID-19 for Black, Hispanic and Asian patients (p<0.001) was reported by one study [41]. Other studies reported that patients of black or Hispanic ethnicity were significantly more likely to complete a telephone consultation over a video consultation compared to patients of White or Asian ethnicity (p<0.001) [38, 44].

#### Language

Language barriers and effect on the uptake of remote consultations were highlighted by nine studies [32, 35, 37, 44, 46, 48, 50, 54, 56]. Adeli found that contacting patients whose first language was not English was problematic, yet once contacted, patients were likely to accept a remote consultation (n = 6/17, 35.3%). Interpreters were used for 50% of remote consultations [32]. Xiong’s USA based study reported that patients whose primary language was not English or Spanish were significantly less likely to use remote consultations compared to those whose primary language was English or Spanish (OR 0.34 [95% CI 0.18 to 0.65]; p = 0.001), [50]. The percentage of patients whose primary language was not English decreased significantly with remote consultations (p<0.001) in the study by Franciosi [46] but Shehan [48] reported no significant differences between the groups in the primary languages spoken. Three studies found that those with a first language other than English were less likely to opt for remote consultations but those that did were more likely to choose a telephone consultation than a video consultation [37, 44, 54].

#### Urban and rural residence

Six studies reported on participants’ urban/rural status and its association with the uptake of remote consultations, four in the USA [31, 39, 41, 47], one in Australia [53], and one in Canada [55]. Four of these studies reported that patients living in an urban area were significantly more likely to use remote consultations compared to those living in a rural location; Jaffe reported that respondents living in urban areas accounted for 92.1% of remote consultations compared to 88.5% of in-person encounters (p = 0.005), [31, 39, 47, 54]. However, Lepage noted that of the 242 patients living in rural areas 17.5% received a remote consultation compared to 0.21% of those living in urban areas [55]. Lonergan et al. reported a significant increase in the use of video visits by those living in urban areas during COVID-19 compared to the pre-COVID-19 period (p<0.001) [41].

### Revisits and non-attendance rates

A small number of patients receiving remote consultations, as reported by two studies, required follow-up face-to-face or emergency visits. Four percent in the study by Sellars et al. [59] and 2.8% in the study by Irarrazaval et al [57] required a face-to-face consultation following their telehealth visit and an emergency visit was required by 1.9% of patients in the latter study. Another study reported no subsequent emergency visits for either remote consultation or usual care patients within 30 days of the consultation and that the revisit rate for both groups was similar [34]. ‘Did not attend’ (DNA) rates were reported by six papers [36, 40, 46, 48, 52, 59]. Shehan reported a non-significant reduction in DNA’s during COVID-19 compared to pre-COVID-19 [48]. In contrast, Franciosi et al reported a significant reduction in non-attendance rates from 12.9% in 2019 to 10.5% in 2020 (p<0.001) [46].

## Discussion

This review was designed to add to the existing evidence by evaluating inequalities in the invitation to and/or uptake of remote outpatient consultations for chronic conditions in secondary and tertiary care. The authors identified 29 studies reporting invitations and/or uptake of remote consultations by patient characteristics, all of moderate to high quality. For those in clinical practice, these findings may help to illuminate the extent to which inequalities matter in regard to remote consultations and to help services understand and address potential barriers. However, most of the included studies reported narrative data focused on the uptake of a remote consultation rather than the invitation to a remote consultation (particularly those where data were extracted from databases and electronic health records), therefore we were unable to report comparative information on the inequalities associated with invitation to a remote consultation specifically.

Uptake of video consultations ranged from 5% to 78% and telephone consultations from 12% to 78%. In line with other studies, this review has found that in general, patients who are older in age; male; have lower household incomes; are unemployed; have lower educational attainment; are from an ethnic minority group, live in a rural location or do not speak English as their first language are less likely to engage with remote consultations [60, 61].These findings may suggest that other factors, or multiple factors, are important in explaining uptake of remote consultations. Intersectional approaches are increasingly recognised as offering insights to inequalities in health outcomes and behaviours [62, 63]. As found in the primary care population certain groups of patients such as those from older age groups when offered a choice, tended to choose a telephone consultation rather than a video consultation [4]. Similarly those of non-white ethnicity were less likely to choose to have a remote consultation but where they did, they were significantly more likely to choose a consultation by telephone rather than by video (p = <0.001).

In the period before COVID-19 the uptake of remote consultations, where offered, was reported to be less than 20% in the included studies. Unsurprisingly, uptake increased substantially during the COVID-19 pandemic but data from the included studies were heterogeneous so it wasn’t possible to synthesise these data or test for differences between the pre- and post-COVID time periods. Clearly the specific context of the pandemic is likely to have changed the benefit/risk equation to uptake of a remote consultation for people needing hospital outpatient care. Other studies have reported a significant increase in remote consultations, for example in the study by Schulz remote consultations increased by 2,255% however, as in certain countries with large rural populations there was already an established telehealth system in place pre-pandemic [64].

The benefits to having a remote consultation for patients in disadvantaged groups include: reduced time and travel costs, have multiple chronic conditions or are unable to take time off work to attend appointments [16]. Nevertheless, there are many barriers preventing these patients from using remote consultations which often affect those of greatest need. Older people may be reluctant or unable to embrace new technologies or new ways of working [12] and are concerned about the confidentiality of remote consultations as are those who live in shared accommodation and have difficulty in finding a private space to have a remote consultation [65]. Patients on low annual household incomes or from disadvantaged groups tend to be digitally excluded compared to those with a higher annual household income and white, and those living in rural areas can have poor digital infrastructure [66, 67].

### Recommendations for policy, research and practice

Despite many countries implementing policies aimed at increasing the use of remote consultations within healthcare [2, 68], this study, along with others, has found there are still inequalities in the uptake of remote consultations [5, 65] particularly in older age groups and groups with protected characteristics as outlined by PROGRESS PLUS. In order to address these disparities thought needs to be given to ways in which inequity in the uptake of remote consultations can be overcome [69].

This research has found certain sectors of society prefer a remote consultation by telephone rather than video however, the findings are not conclusive. To help inform future practice, research needs to be undertaken to understand which mode of remote consultation (telephone or video) is preferred by different sectors of society [4].

We recommend that when implementing remote consultations in secondary or tertiary care policy makers need to take the above potential barriers around patients’ social and cultural contexts into consideration to mitigate inequalities in the uptake of remote consultations [70]. In order to achieve this it has been suggested that potential service users be involved in the implementation of remote consultations, and that clear messages about remote consultations to different patient groups should be delivered and most importantly that services need to be tailored to take into account the needs of different groups [69]. These could include providing the use of local hubs for those who live in shared spaces where confidentiality can be an issue or for those who do not have access to appropriate technology thereby helping to mitigate digital exclusion [71]. Overall, flexibility in the design and delivery of remote consultations which reflect the cultural needs of different disadvantaged groups may be required as one size does not appear to fit all [72, 73]. Finally it should not be forgotten that for many patients a remote consultation may not be appropriate and others prefer to speak to their physician in-person so a remote consultation should always be an option rather than mandatory [65, 71].

### Strengths and limitations

Whilst we attempted to be as inclusive as possible in the choice of databases searched for this review, we did not explicitly search specific databases such as the Cochrane Central Register of Controlled Trials. This may have resulted in relevant interventional studies being overlooked in our search results. However, as none of the studies included in this review was a clinical trial, this is unlikely to have impacted on our findings. Limiting the search to the English language may introduce language bias and have an effect on the precision, generalisability and applicability of review results [74]. However, it has also been argued that omitting non-English language studies does not alter the findings or conclusions of most systematic reviews [75]. We were unable to report on all the data and analyses outlined in the protocol because some of this data was not reported in the studies. The studies included in this review reported data on the uptake of remote consultations but there was a lack of data relating to rates of offer/invitation to remote consultations. Although not in our original plan for the review, as some studies included data on non-attendance and re-visit rates, we included this information as it may have implications for the clinical efficiency of remote consultations. Additionally, there was substantial heterogeneity in the data preventing us from pooling data in meta-analyses and making it difficult to draw definitive conclusions.

## Conclusion

To conclude, in studies that compared two time periods, most studies reported an increase in the uptake of remote consultations over time. Younger patients were significantly more likely to use video consultations compared to older patients (p = <0.001) and females were more likely to use remote consultations than males. Additionally, it was more likely for females to have video consultations compared to males (p = 0.0013). Patients with ethnicities other than white were less likely to have a remote consultation as were those whose first language is not English (p = <0.001). Of the studies reporting on place of residence most reported that patients living in urban areas accounted for the majority of remote consultation appointment, up to 92.1% in one study.

Although showing an increase in uptake of remote consultations our findings also indicate that remote consultations may still perpetuate or exacerbate existing health inequalities in access to and the uptake of healthcare for some patient populations. Consequently, there is a need for more research on all sociodemographic characteristics which can influence the uptake of digital remote consultations and to determine what works well for different patient groups and why. Effective processes to promote remote consultations where appropriate and to ameliorate health inequalities can then be designed and implemented.

## Supporting information

S1 File Search strategies. (DOCX)

S2 File Example data extraction form. (DOCX)

S3 File Mixed Methods Appraisal Tool (MMAT). (DOCX)

S4 File PRISMA checklist. (DOCX)

S5 File Quality of included studies using MMAT. (DOCX)

S6 File Characteristics and findings of included studies. (DOCX)
