# Supplementary material for: Real-time remote outpatient consultations in secondary and tertiary care: A systematic review of inequalities in invitation and uptake
Source: PLoS One. 2022 Jun 3;17(6):e0269435. doi: 10.1371/journal.pone.0269435 (PMC9165897; doi:10.1371/journal.pone.0269435)
Supplement: S1 File — (DOCX) [file pone.0269435.s001.docx]

**S1 File:**

**Medline search strategy**

1. (teleconsult* or tele-consult*).ti,ab. (1192)
2. (telehealth* or telehealth*).ti,ab. (3992)
3. (telemedicine or tele-medicine).ti,ab. (9847)
4. (telenurs* or tele-nurs*).ti,ab. (171)
5. (video conference* or videoconference*).ti,ab. (2551)
6. “video consultation”.ti,ab. (121)
7. “video visit”.ti,ab. (29)
8. “remote consultation”.ti,ab. (158)
9. (“telephone based” or “phone based” or “text message” or text-message).ti,ab. (3521)
10. eHealth.ti,ab. (2069)
11. mHealth.ti,ab. (2001)
12. “digital health”.ti,ab. (1034)
13. 1 or 2 or 3 or 4 or 5 or 6 or 7 or 8 or 9 or 10 or 11 or 12 (23367)
14. (socioeconomic status or socio-economic status).ti,ab. (42396)
15. Exp Healthcare disparities/ (18292)
16. 14 or 15 (59402)
17. (access* or accept*).ti,ab. (796824)
18. Uptake.ti,ab. (326190)
19. 17 or 18 (1109584)
20. Exp “Referral and Consultation”/ (77045)
21. Invitation.mp. (4068)
22. Exp “Appointments and Schedules”/ (20901)
23. 20 or 21 or 22 (100131)
24. Exp Secondary Care/ or exp Outpatient Clinics, Hospital/ (17870)
25. Exp Tertiary Healthcare/ (1184)
26. Exp Tertiary Care Centers/ (15264)
27. Exp Ambulatory Care/ or exp Outpatients/ (69045)
28. 24 or 25 or 26 or 27 (100634)
29. 16 or 19 or 23 or 28 (1332273)
30. 13 and 29 (9389)

Limit 30 to (English language and yr=”2010-Current”) (8317)

**Embase search strategy**

1. (teleconsult* or tele-consult*).ti,ab. (1886)
2. (telehealth* or tele-health*).ti,ab. (7312)
3. (telemedicine or tele-medicine).ti,ab. (17054)
4. (tele-nurs* or tele-nurs*).ti,ab. (222)
5. (video conference* or videoconference*).ti,ab. (3811)
6. “video consultation*.ti,ab. (286
7. “video visit”.ti,ab.(115)
8. “remote consultation”.ti,ab. (287)
9. (“telephone based” or “phone based” or “text message” or text-message).ti,ab. (6389)
10. eHealth.ti,ab. (3362)
11. mHealth.ti,ab. (3626)
12. “digital health”.ti,ab. (2370)
13. 1 or 2 or 3 or 4 or 5 or 6 or 7 or 8 or 9 or 10 or 11 or 12 (41272)
14. (“socioeconomic status” or “socio-economic status”).ti,ab. (65073)
15. Exp health care disparity/ (17018)
16. 14 or 15 (81074)
17. (access* or accept*).ti,ab. (1323317)
18. Uptake.ti,ab. (464442)
19. 17 or 18 (1764296)
20. Exp patient referral/ (123293)
21. Appointment.ti,ab. (26985)
22. Invitation.ti,ab. (7239)
23. 20 or 21 or 22 (154804)
24. Secondary health care/ (7132)
25. Exp outpatient care/ (38501)
26. Exp tertiary heath care/ (110220)
27. Exp ambulatory care/ (49483)
28. 24 or 25 or 26 or 27 (202368)
29. 16 or 19 or 23 or 28 (2139782)
30. 13 and 29 (15621)
31. Limit 30 to (english language and yr=”2010-current”) (14923)
32. Limit 31 to (adult <18 to 64 years> or aged <65+ years>) (7337)

**Applied social sciences index and abstracts (ASSIA) search strategy**

[(noft(teleconsult* OR tele-consult* OR telehealth* OR tele-health* OR telemedicine OR tele-medicine) OR noft(telenurs* OR tele-nurs* OR video conferenc* OR videoconferenc* OR video consultation OR video visit OR remote consultation) OR noft(telephone based OR phone based OR text message OR text-message OR eHealth OR digital health)) AND ((noft(socioeconomic status OR socio-economic status) OR noft(healthcare disparities OR health inequalities)) OR (noft(access* OR accept*) OR noft(uptake)) OR (noft(referral OR consultation) OR noft(invitation) OR noft(appointment*)) OR (noft(tertiary healthcare OR tertiary care centre*) OR noft(tertiary healthcare OR tertiary care centre*) OR noft(ambulatory care OR outpatient*))) AND (la.exact("ENG") AND yr(2010-2029))](https://www.proquest.com/myresearch/savedsearches.checkdbssearchlink:rerunsearch/1918559/SavedSearches?site=assia&t:ac=SavedSearches) (2911)

**HMIC search strategy**

1. teleconsult*.mp. or tele-consult*.ti,ab. [mp=title, other title, abstract, heading words]
2. telehealth*.mp. or tele-health*.ti,ab. [mp=title, other title, abstract, heading words]
3. telemedicine.mp. or tele-medicine.ti,ab. [mp=title, other title, abstract, heading words]
4. telenurs*.mp. or tele-nurs*.ti,ab. [mp=title, other title, abstract, heading words]
5. video conferenc*.mp. or teleconferenc*.ti,ab. [mp=title, other title, abstract, heading words]
6. video consultation.ti,ab.
7. remote consultation.ti,ab.
8. video visit.ti,ab.
9. (telephone based or phone based or text message).mp. or text-message.ti,ab. [mp=title, other title, abstract, heading words]
10. eHealth.ti,ab.
11. digital health.ti,ab.
12. 1 or 2 or 3 or 4 or 5 or 6 or 7 or 8 or 9 or 10 or 11
13. socioeconomic status.mp. or socio-economic status.ti,ab. [mp=title, other title, abstract, heading words]
14. Healthcare disparities.mp. or exp Health inequalities/
15. exp Socioeconomic factors/
16. 13 or 14 or 15
17. access*.mp. or accept*.ti,ab. [mp=title, other title, abstract, heading words]
18. uptake.ti,ab.
19. 17 or 18
20. exp Referral rates/ or exp Referral letters/ or exp Referral/ or exp Patient referral/ or exp Referral patterns/
21. consultation.mp.
22. invitation.mp.
23. appointments.mp. or exp Patient appointments/
24. 20 or 21 or 22 or 23
25. secondary care.mp. or exp hospital care/
26. exp Out patient departments/ or outpatient clinics.mp. or exp Out patient services/
27. tertiary healthcare.mp.
28. tertiary care cent*.mp.
29. exp Ambulatory care/
30. exp Out patients/
31. 25 or 26 or 27 or 28 or 29 or 30
32. 16 or 19 or 24 or 31
33. 12 and 32
34. limit 33 to (yr="2010 -Current" and English)

**PsycINFO search strategy**

1. teleconsult*.mp. or tele-consult*.ti,ab. [mp=title, abstract, heading word, table of contents, key concepts, original title, tests & measures, mesh]
2. telehealth*.mp. or tele-health*.ti,ab. [mp=title, abstract, heading word, table of contents, key concepts, original title, tests & measures, mesh]
3. telemedicine.mp. or tele-medicine.ti,ab. [mp=title, abstract, heading word, table of contents, key concepts, original title, tests & measures, mesh]
4. telenurs*.mp. or tele-nurs*.ti,ab. [mp=title, abstract, heading word, table of contents, key concepts, original title, tests & measures, mesh]
5. video conferenc*.mp. or videoconferenc*.ti,ab. [mp=title, abstract, heading word, table of contents, key concepts, original title, tests & measures, mesh]
6. video consultation.ti,ab.
7. video visit.ti,ab.
8. remote consultation.ti,ab.
9. (telephone based or phone based or text message).mp. or text-message.ti,ab. [mp=title, abstract, heading word, table of contents, key concepts, original title, tests & measures, mesh]
10. eHealth.ti,ab.
11. digital health.ti,ab.
12. 1 or 2 or 3 or 4 or 5 or 6 or 7 or 8 or 9 or 10 or 11
13. socioeconomic status.mp. or socio-economic status.ti,ab. [mp=title, abstract, heading word, table of contents, key concepts, original title, tests & measures, mesh]
14. exp Health Disparities/ or exp "Racial and Ethnic Differences"/ or Healthcare disparities.mp.
15. 13 or 14
16. access*.mp. or accept*.ti,ab. [mp=title, abstract, heading word, table of contents, key concepts, original title, tests & measures, mesh]
17. uptake.ti,ab.
18. 16 or 17
19. referral.mp. or consultation.ti,ab. [mp=title, abstract, heading word, table of contents, key concepts, original title, tests & measures, mesh]
20. invitation.mp.
21. appointments.mp. or schedules.ti,ab. [mp=title, abstract, heading word, table of contents, key concepts, original title, tests & measures, mesh]
22. 19 or 20 or 21
23. secondary care.mp.
24. exp Outpatient Treatment/ or exp Outpatients/ or outpatient clinics.mp.
25. tertiary healthcare.mp.
26. tertiary care centre.mp.
27. ambulatory care.mp. or exp Outpatient Treatment/
28. 23 or 24 or 25 or 26 or 27
29. 15 or 18 or 22 or 28
30. 12 and 29
31. limit 30 to (english language and yr="2010 -Current")

**CINAHL search strategy**

1. “teleconsult* or tele-consult*” or (MH “remote consultation”)
2. (MH “telehealth”) or “telehealth* or telehealth*”
3. “telemedicine or tele-medicine”
4. (MH “telenursing”) or “telenurs* or “tele-nurs*”
5. (MH “videoconferencing”) or (MH “teleconferencing”) or “video conferenc* or videoconference*”
6. “video consultations or video appointments”
7. “video visit”
8. (MH “text messaging”) or “telephone-based or “phone-based” or “text messag*”
9. “eHealth”
10. “mHealth or mobile health or m-health”
11. *“*digital health or digital healthcare or digital health interventions or digital health care”
12. (MH “internet-based intervention”)
13. S1 or S2 or S3 or S4 or S5 or S6 or S7 or S8 or S9 or S10 or S11 or S12
14. (MH “socioeconomic factors”) or (MH “health status disparities”) or “socioeconomic status”
15. (MH “healthcare disparities)
16. S14 or S15
17. (MH (“health services accessibility”) or “access to health care”
18. “acceptability”
19. “uptake or motivations or barriers to uptake or adherence”
20. S17 or S18 or S19
21. (MH “referral and consultation”)
22. “invitation”
23. (MH “appointments and schedules”)
24. S21 or S22 or S23
25. (MH “secondary health care”)
26. (MH “outpatient service”) or (MH “ambulatory care facilities”) or (MH “outpatients”) or (MH “ambulatory care”)
27. (MH “tertiary health care”)
28. “tertiary care center”
29. S25 or S26 or S27 or S28
30. S16 or S20 or S24 or S32
31. S13 and S30 (limiter year 2010-2021) (3619)
