# Supplementary material for: Real-time remote outpatient consultations in secondary and tertiary care: A systematic review of inequalities in invitation and uptake
Source: PLoS One. 2022 Jun 3;17(6):e0269435. doi: 10.1371/journal.pone.0269435 (PMC9165897; doi:10.1371/journal.pone.0269435)
Supplement: S2 File — (DOCX) [file pone.0269435.s002.docx]

**S2 File: Data extraction form**

Reviewer name:

Date:

| **Identifying features** | | | | |
| --- | --- | --- | --- | --- |
| Reference ID number |  | | | |
| Citation |  | | | |
| **Study characteristics** | | | | |
| Where research was conducted (country) |  | | | |
| Data collection period |  | | | |
| Healthcare setting (i.e. secondary or tertiary care) |  | | | |
| Health condition |  | | | |
| Study design |  | | | |
| No. of participants recruited |  | | | |
| No. of participants dropping out |  | | | |
| **Methodological characteristics** | | | | |
| Overarching aim of the study |  | | | |
| Research question(s) |  | | | |
| Data collection methods |  | | | |
| Approach to data analysis |  | | | |
| **Interventions** |  | | | |
| Type of intervention (i.e. teleconsultation) |  | | | |
| Comparator (i.e. usual care) |  | | | |
| Total No of participants approached |  | | | |
| **Participant characteristics** | **In-person 2019**  **n (%)** | **Telemedicine 2020**  **n (%)** | **P value** |  |
| No of participants offered |  |  |  |  |
| No of participants taking up the offer |  |  |  |  |
| Area of residence: |  |  |  |  |
| - Urban |  |  |  |  |
| - Rural |  |  |  |  |
| *Progress characteristics:* |  |  |  |  |
| Age groups |  |  |  |  |
| - Mean (range) |  |  |  |  |
|  |  |  |  |  |
| Gender - Male |  |  |  |  |
| Gender – Female n (%) |  |  |  |  |
|  |  |  |  |  |
| Ethnicity: n (%) |  |  |  |  |
| - Asian |  |  |  |  |
| - Black |  |  |  |  |
| - Hispanic/Latino |  |  |  |  |
| - White/Caucasian |  |  |  |  |
| - Other |  |  |  |  |
| - Unknown |  |  |  |  |
| Household income: |  |  |  |  |
| - Mean (range) |  |  |  |  |
|  |  |  |  |  |
| Language: n (%) |  |  |  |  |
| - English |  |  |  |  |
| - Spanish |  |  |  |  |
| - Other |  |  |  |  |
| - Unknown |  |  |  |  |
|  |  |  |  |  |
| Socio-economic status (please state level and numbers) |  |  |  |  |
| Note any other Progress-Plus factors described in the paper (i.e. disabilities, religious beliefs, language barriers, need for interpreters) | | | | |
| **Outcomes and findings** |  | | | |
| Outcomes including:   - The number of participants experiencing problems with the mode of delivery - The number of participants who required a follow up F2F appointment after their remote consultation |  | | | |
| Note any examples of good practice or continuity of care (if reported) |  | | | |
| Limitations |  | | | |
| Author’s study summary and conclusions |  | | | |
| Linked publications (please record any publications linked to this one and provide the citation) |  | | | |
| Notes (please record any additional notes, thoughts or ideas |  | | | |
| **Quality assessment MMAT score** |  | | | |
