# Supplementary material for: Real-time remote outpatient consultations in secondary and tertiary care: A systematic review of inequalities in invitation and uptake
Source: PLoS One. 2022 Jun 3;17(6):e0269435. doi: 10.1371/journal.pone.0269435 (PMC9165897; doi:10.1371/journal.pone.0269435)
Supplement: S5 File — (DOCX) [file pone.0269435.s005.docx]

**S5 File: Quality assessment by MMAT**

| **Quantitative non-randomized studies** | **3.1** | **3.2** | **3.3** | **3.4** | **3.5** |
| --- | --- | --- | --- | --- | --- |
| Abel | Yes | Yes | Yes | Yes | Yes |
| Adeli | Can’t tell | Yes | Yes | Yes | Yes |
| Andino | Yes | Yes | Yes | Yes | Yes |
| Chunara | Yes | Yes | No | Yes | Yes |
| Darrat | Yes | Yes | Can’t tell | Yes | Yes |
| Eberly | Yes | Yes | Yes | Yes | Yes |
| Franciosi | Yes | Yes | Yes | Yes | Yes |
| Gilson | Yes | Yes | No | Yes | Yes |
| Irarrazaval | Yes | Yes | Yes | Yes | Yes |
| Lepage | Yes | Yes | Yes | Yes | Yes |
| Liu | Yes | Yes | Yes | Yes | Yes |
| Lonergan | Yes | Yes | Yes | No | Yes |
| Ohlstein | Yes | Yes | Yes | Yes | Yes |
| Poeran | Can’t tell | Yes | Yes | Yes | Yes |
| Rodriguez | Yes | Yes | Yes | Yes | Yes |
| Rowe | Yes | Yes | Yes | Yes | Yes |
| Sellars | Yes | Yes | Yes | Yes | Yes |
| Stevens | Yes | Yes | No | Yes | Yes |
| Wegerman | Yes | Yes | Can’t tell | Yes | Yes |
| Xiong | Yes | Yes | Yes | Yes | Yes |
| Yuan | Yes | Yes | Yes | Yes | Yes |
| **Quantitative descriptive** | **4.1** | **4.2** | **4.3** | **4.4** | **4.5** |
| Almandoz | Yes | No | Yes | Yes | Yes |
| Jaffe | Yes | Yes | Yes | No | Yes |
| Kemp | Yes | Yes | Yes | Yes | Yes |
| Menon | Yes | Can’t tell | Yes | Can’t tell | Yes |
| Santonicola | Yes | Yes | Yes | No | Yes |
| Shehan | Yes | Yes | Yes | Yes | Yes |
| **Mixed methods** | **5.1** | **5.2** | **5.3** | **5.4** | **5.5** |
| Lewis | Yes | Yes | Yes | Yes | Yes |
| Moo | Yes | Yes | Yes | Yes | Yes |

**Key:** **3.1.** Are the participants representative of the target population? **3.2.** Are measurements appropriate regarding both the outcome and intervention? **3.3.** Are there complete outcome data? **3.4**. Are the confounders accounted for in the design and analysis? **3.5.** During the study period, is the intervention administered as intended? **4.1.** Is the sampling strategy relevant to address the research question? **4.2.** Is the sample representative of the target population? **4.3.** Are the measures appropriate? **4.4.** Is the risk of nonresponse bias low? **4.5.** Is the statistical analysis appropriate to answer the research question? **5.1.** Is there an adequate rationale for using a mixed methods design to address the research question? **5.2.** Are the different components of the study effectively integrated to answer the research question? **5.3.** Are the outputs of the integration of qualitative and quantitative results adequately addressed? **5.4**. Are divergences and inconsistencies between quantitative and qualitative results adequately addressed? **5.5.** Do the different components of the study adhere to the quality criteria of each tradition of the methods involved?
