# Supplementary material for: Real-time remote outpatient consultations in secondary and tertiary care: A systematic review of inequalities in invitation and uptake
Source: PLoS One. 2022 Jun 3;17(6):e0269435. doi: 10.1371/journal.pone.0269435 (PMC9165897; doi:10.1371/journal.pone.0269435)
Supplement: S6 File — (DOCX) [file pone.0269435.s006.docx]

**S6 File: Characteristics and findings of the included studies**

| **Author, Year (Country), Condition(s), Setting** | **Design, Source, Time period** | **Intervention (n), Comparator (n), Dropouts (n), Total** | **Findings** |
| --- | --- | --- | --- |
| Abel 2018 (USA)  Mental health  Tertiary care | RC  D/b  01/10/2007 to 31/03/2012 | **I:** 1) MHV – MyHealthVet personal health record, (n=499,445)  2) patient portal and CVT – Clinical Video Telehealth, (n=81,939)  3) Both, (n=32,723)  **C:** Usual care, (n=1,557,218) | **AGE:** MHV: Mean age (SD) =56.2 (14.7). <40=81,624, 40-59=179,169, 60-79=213,008, ≥80=25,644. CVT: Mean age (SD =57.0 (14.7). <40=12,632, 40-59=27,136, 60-79=37,585, ≥80=4,586. Both: Mean age (SD) =53.1 (13.7). <40=6,712, 40-59=12,770, 60-79=12,696, ≥80=545. UC: Mean age (SD) =61.7 (16.0). <40= 76,172, 40-59=434,843, 60-79=721,951, ≥80=224,252.  **GENDER:** MHV: Male=435,459, Female=63,986. CVT: Male=75,733, Female=6,206. Both: Male=28,509, Female=4,214. UC: Male=1,447,141, Female=110,077.  **ETHNICITY:** MHV: Asian=N/A, Black=65,704, Hispanic/Latino=2048, White=373,057, Other=14,533. CVT: Asian=N/A, Black=10,206, Hispanic/Latino=445, White=61,208, Other=2,669. Both: Asian=N/A, Black=3116, Hispanic/Latino=114, White=26,075, Other=943. UC: Asian=N/A, Black=285,081, Hispanic/Latino=10,206, White=1,061,488, Other=41,732  **SES:** MHV: High economic need=98,779 (19.8%). CVT: High economic need=20,545 (25.1%). Both: High economic need=5746 (17.6%). UC: High economic need=442,658 (28.4).  **URBAN/RURAL:** MHV: Urban=382,066, Rural=106,920 CVT: Urban=40,298, Rural=40,342. Both: Urban=27,954, Rural=14,256. UC: Urban=1,135,344, Rural=384,237 |
| Adeli 2021 (USA)  Opthalmology  Tertiary care | CS  EHR  01/02/2020 to 01/06/2020 | **I:** TM (n=6)  **C:** UC (n=11)  Dropouts (n=1)  Total n=18 | **AGE:** TM: 53.5±15.2 (31-72) UC: 48.5±13.2 (34-82)  **GENDER:** TM: Male=4(66.7%), Female=2(33.3%). UC: Male=5(45.5%), Female=6(54.5%).  **ETHNICITY:** TM: Black=3, Hispanic/Latino=2, Other=1. UC: Black=5, Hispanic/Latino=6.  **LANGUAGE:** TM: 1/6 English. Others: Amharic (n=1), Farsi (n=1), Spanish (n=2), Twi (n=1). Interpreters used in 3/6 telephone appointments. UC: 2/11 English. Others: Fulani (n=1), Somali (n=1), Spanish (n=6), Twi (n=1). |
| Almandoz 2021 (USA)  Obesity  Tertiary care | RR  RV  01/06/2020 to 24/09/2020 | **I:** TH (n=408)  **C:** IP (n=175)  Total (n=787) | **AGE:** TH: <45=117, 45-64=203, ≥65=86. Mean age=52.3 (12.9). IP: <45=41, 45-64=74, ≥65 = 60. Mean age=56.3 (13.5). *Mean age of patients using telehealth was significantly lower than those having IP appointments* ***p=0.001.***  **GENDER:** TH: Male=67, Female=335. IP: Male=24, Female=150.  **ETHNICITY:** TH: Black=97, Hispanic/Latino=73, White=203, Other=17. IP: Black=26, Hispanic/Latino=30, White=99, Other=8.  **EDUCATION:** TH: High school = 40, some college/technical school = 141, College graduate = 224. IP: High school = 14, some college/technical school = 61, College graduate = 100.  **INCOME:** TH: Low income (<25k) = 54 (13.5%); highest income (>75k) = 189 (47.1%). IP: low income = 13 (7.5%); highest income = 95 (54.9%). |
| Andino 2020 (USA)  Urology  Tertiary care | MC  EHR  11/07/2016 to 04/02/2020 | **I:** V (n=600)  **C:** UC (n=600)  Total (n=1200) | **AGE:** V: Mean (range) 51 (36 to 62). UC: Mean (range) 61 (45 to 71). UC *significantly older* ***p=<0.0001.***  **GENDER:** V: Male=382 (64%), Female=218 (36%). UC: Male=434 (72%), Female=166 (28%). *Women significantly more likely to use* *Video* ***p=0.0013***. |
| Chunara 2021 (USA)  COVID-19  Tertiary care | C  EHR  19/03/2019 to 30/04/2019 vs. same period in 2020 | **I:** TM (n=900 in 2019; n=90991 in 2020)  **C:** UC (n=331793 in 2019; 36901 in 2020)  Total (n=460,585) | **AGE:** UC in 2020: Mean (SD) =48.6(23.5). UC in 2019: Mean (SD) =51.0(23.3). TM in 2020: Mean (SD) =47.3(19.3). TM in 2019: Mean (SD) =40.7(13.7).  **GENDER:** UC in 2020: Male=44.2%, Female=55.8%. UC in 2019: Male=41.7%, Female=58.3%. TM in 2020: Male=39.3%, Female=60.7%. TM 2019: Male=33.3%, Female=66.7%.  **ETHNICITY:** UC in 2020: Asian=5.8%, Black=16.9%, Hispanic/Latino=1.3%, White=47.9%, Other=15.3%, Multiple=1.1%, Unknown=11.7%. UC in 2019: Asian=4.7%, Black=10.4%, Hispanic/Latino=1.2%, White=61.5%, Other=9.3%, Multiple=1.1%, Unknown=11.7%. TM in 2020: Asian=5.3%, Black=12.9%, Hispanic/Latino=2.0%, White=51.1%, Other=12.4%, Multiple=1.7%, Unknown=14.7%. TM in 2019: Asian-6.6%, Black=8%, Hispanic/Latino=3.2%, White=53.3%, Other=9.3%, Multiple=3%, Unknown=16.6%  **LANGUAGE:** UC in 2020: English=73.5%, Spanish=15.3%. UC in 2019: English=87.36%, Spanish=5.7%. TM in 2020: English=89.7%, Spanish=3.7%. TM in 2019: English=92.3%, Spanish=1% |
| Darrat 2021 (USA)  Otolaryngology  Tertiary care | RC  EHR  17/03/2020 to 01/05/2020 | **I:** T (n=144) or V (n=437)  **C:** IP (n=409)  **DNA:** (n=172)  Total (n=1,162) | **AGE:** T: Median=62 (0-90). V: Median=49 (0-89). UC: Median=57 (0-97). DNA: Median=52 (0-95).  **GENDER:** T: Male=78 (54.2%), Female=66 (45.8%). V: Male=190 (43.5%), Female=247 (56.5%). UC: Male=205 (50.1%), Female=204 (49.9%). DNA: Male=85 (49.4%), Female=87 (50.6%).  **ETHNICITY:** T: Black=38(26.4%), White=80(55.6%), Other=26(18.1%). V: Black=110(25.2%), White=254 (58.1%), Other=73(16.7%). UC: Black=82(20%), White=275(67.2%), Other=52(12.7%). DNA: Black=59(34.3%), White=90 (52.3%), Other=23(13.4%). |
| Eberly 2020 (USA)  Cardiology, pulmonology, rheumatology, gastroenterology, infectious diseases, nephrology, haematology-oncology  Secondary care | RC  EHR  16/03/2020 to 11/05/2020 | **I**: T (n= 18690) or V (n=17607)  **C:** DNA telemedicine appointment (n=34663)  Total (n=70,960) | **AGE:** T: <55=5572(29.8%), 55-64=4242(22.7%), 65-74=4797(25.7%), ≥75=4079(21.8). V: <55=7176(40.8%), 55-64=3957(22.5%), 65-74=4110(23.3%) ≥75=2364(19.7%). DNA: <55=11,665(33.7%), 55-64=7668(22.1%), 65-74=8517(24.6%), ≥75=6813(19.7%). *Younger patients significantly more likely to have V than older patients* ***(p<0.001)*** *and significantly more likely to have completed a remote consultation than older patients* ***(p<0.001).***  **GENDER:** T: Male=8390(44.9%), Female=10,300(55.1%). V: Male=8242(46.8%), Female=9365 (53.2%). DNA: Male=14,776(42.6%), Female=19,887(57.4%). *Females significantly more likely to have telephone than video consultation* ***(p<0.001, OR 0.92 [95% CI 0.90-0.95])*.**  **ETHNICITY:** T: Asian=490(2.6%), Black=4257(22.8%), Hispanic/Latino=710(3.8%), White=11,936(63.9%), Other=486(2.6%), Unknown=811(4.3%). V: Asian=574(3.3%), Black=2683(15.2%), Hispanic/Latino=598(3.4%), White=1243(70.5%), Other=463(2.6%), Unknown=877 (55). DNA: Asian=1395(4%), Black=5828(16.8%), Hispanic/Latino=1369(3.9%), White=23,213(67%), Other=1009(2.9%), Unknown=1737(5%).  **LANGUAGE:** T: English Language=18,218(97.5%). V: English Language=17,348(98.5%). DNA: English Language=33,141(96.4%). *English speakers significantly more likely to have V than T consultation (****p<0.001****) and significantly more likely to complete any remote consultation (****p<0.001****).* |
| Franciosi 2021  (USA)  Any  Secondary care | CS  EHR  May and June 2019 compared with May and June 2020. | **I:** T in 2020 (35,234)  **C:** IP 2019 (19,740)  Total (n=54,974) | **AGE:** T: Mean (SD)=55(21). IP: Mean (SD)=57(21). *Patients were significantly younger in the T group* ***p<0.001***  **GENDER:** T: Male=8,509(43.1%). Female=11,231(56.9%). IP: Male=15,839(45%). Female=19,395(55%).  **ETHNICITY**: T: Asian=553(2.8%), Black=1009(5.1%), Hispanic/Latino 2453 (12.4%), White=14995(76%), Other=562(2.8%), Unknown=168 (0.9%). IP: Asian=1022(2.9%), Black=1791(5.1%), Hispanic/Latino=4266(12.1%), White=26661(75.7%), Other=1008(2.9%), Unknown=485(1.4%). *The % of white patients increased significantly with T* ***(p<0.001).***  **LANGUAGE:** T: English language 2020=17935(90.9%). Non-English 2020=1799(9.1%). IP: English language 2019=31427(89.2%). Non-English 2019=3791(10.8%). *Those whose primary language is not English were significantly less likely to have Tcompared to those whose first language is English* ***p<0.001****.*  **NON-ATTENDANCE:** *Significant reduction in non-attendance rates from 12.9% in 2019 to 10.5% in 2020* ***(p<0.001)*** |
| Gilson 2020 (USA)  Any  Tertiary care | RC  EHR  15/03/2020 to 31/05/2020 compared to same period in 2019 | **I:** T (n=18814) or V (n=29661)  **C:** IP (n=31606)  Total (n=80,081) | **AGE:** T: 18-45=3507(18.6%), 46-64=5677(30.2%), 64-74=4587(24.4%), ≥75=4489(23.9%). V: 18-45=9687(32.7%), 46-64=8151(27.5%), 64-74=4596(15.5%), ≥75=2633(8.8%). IP: 18-45=8192(25.9%), 46-64=8455(26.8%), 64-74=5957(18.8%), ≥75=4065(12.9%). *Increasing age associated with a greater likelihood of T rather than V consultation (****p<0.001****).*  **GENDER:** T: Male=7043(37.4%), Female=11,771(62.6%). V: Male=11,290(38.1%), Female=18,371(61.9%). IP: Male=13,177(41.7%), Female=18,429(58.3%).  **ETHNICITY:** T: Black=10,064(53.4%), White=7,084(37.7%), Other=1,666(8.9%). V: Black=10,647(35.9%), White=14,811(49.9%), Other=4,203(14.2%). IP: Black=14,141(44.7%), White=14,112(44.7%), Other=3,353(10.6). *Black patients significantly less likely to have V than T consultations in comparison to white patients (****p<0.001*** *0.55 95%CI: 0.52 to 0.57).* |
| Irrazaval 2020 (Chile)  Gastrointestinal surgery  Secondary care | P  D/b  15/03/2020 to 19/07/2020 | **I:** V (n=113)  **C:** IP (n=106)  Total (n=219) | **AGE:** V: Mean: 49(SD+14.1). IP: Mean: 53(SD+20).  **GENDER:** V: Male=48(45%), Female=58(55%). IP: Male=54(48%), Female=59(52%).  **RE-VISITS:** V: Three (2.8%) patients required a subsequent in-person visit; Two (1.9%) visited the ED within 30 days of appointment. Sixteen (14.9%) patients had a subsequent follow-up appointment within 30 days after surgery. UC: Four patients (3.5%) had second visit (2.8% vs 3.5%; **p=0.09**); seven patients (6.2%) visited the ED. |
| Jaffe 2020 (USA)  Any  Secondary care | RC  IC  March 2019 and March 2020 | **I:** TH (n=669)  **C:** IP (n=10945)  Total (n=11,614) | **AGE:** TH: Mean age=48.8(SD 16.9). 18-44=43.3%, 45-64=35.6%, ≥65=21.1%. IP: Mean age=54.9(SD 16.3), 18-44=28.8%, 45-64=38.9%, ≥65=32.5%. *Younger age groups more likely to have a TH compared to older patients* ***p=<0.001****.*  **GENDER:** TH: Male=29%, Female=71%. IP: Male=34.7%, Female=65.3%. *Females more likely to use TH* ***p<0.002.***  **ETHNICITY:** TH: Asian=3%, Black=9.3%, Hispanic/Latino=9.9%, White=81.8%, Other=6%. IP: Asian=4.8%, Black=9.6%, Hispanic/Latino=7.4%, White=80.2%, Other=5.4%.  **EDUCATION:** TH: Degree=58.4%. IP: Degree=51.8%.  **INCOME:** TH: ≥$75k=35%, <$75k=58%, unknown=7%. IP: ≥$75k=38%, <$75k=55.5%, unknown=6.4%.  **EMPLOYMENT:** TH: Employed=58.4%. P: Employed=51.8% *TH uptake more likely in those in employment compared to the unemployed* ***p<0.001.***  **URBAN/RURAL:** TH: Urban=92.1%, Rural=7.9%. IP: Urban=88.5%, Rural=11.5% *Patients living in urban areas more likely to have a TH visit compared to those living in rural areas* ***p=0.005.*** |
| Kemp 2020 (USA)  Abdominal surgery  Tertiary care | RC  EHR  January 2019 to June 2019 | **I:** Completed e-visit (n=156)  **C:** Non-completion of e-visit (n=43)  Total = (n=199) | **AGE:** eVisit: 18-29=32(20.5%), 30-39=37(23.7%), 40-49=35(22.4%), 50-59=30(19.2%), 60-69=13 (8.3%), 70-79=74.5%), ≥80 =2(1.3%). Non-completion eVisit: 18-29=10(23.3%), 30-39=12(27.9%), 40-49=9(20.9%), 50-59=3(7.0%), 60-69=4(9.3%), 70-79 4(9.3%), ≥80= (2.3%).  **GENDER:** eVisit: Male=61(39.1%), Female=95(60.9%). Non-completion: Male=1637.2%), Female=27(62.8%).  **ETHNICITY:** eVisit: Asian=2(1.3%), Black=12(7.7%), White=136(87.1%), Other=4(2.6%), Unknown=2(1.3%). Non-completion: Asian=3(7.0%), Black=7(16.3%), White=32(74.4%), Other=1(2.3%), Unknown=0(0%). *White patients significantly more likely to complete eVisits.* ***p=0.041.*** |
| Lepage 2020 (Canada)  Hepatitis C  Secondary care | RC  EHR  01/01/2012 to 31/12/2016 | **I:** TM (n=106)  MD (n=81)  **C:** IP (n=1,267)  Total (n=1,454) | **Gender:** TM: Male=72(67.9%), Female=34(32.1%). MD: Male=53(65.4%), Female=28(34.6%). IP: Male=804(63.8%), Female=463(36.2%)  **Ethnicity:** TM: Asian=0(0%), Black=2(1.9%), Hispanic/Latino=0(0%), White=74(69.8%), Indigenous=11(10.4%), Other=1(0.9%). MD: Asian=1(1.2%), Black=1(1.2%), Hispanic/Latino=00%), White =66(81.5%), Indigenous=3(3.7%), Other=1(1.2%). IP: Asian=90(7.1%), Black=96(7.6%), Hispanic/Latino=1(0.08%), White=877(69.2%), Indigenous=27(2.1%), Other=20(1.6%).  **Education:** TM: <High school=18(17.3%). MD: <High school=11(14.1%). IP: <High school=132(10.7%).  **SES:** TM: Greatest material deprivation=33(31.1%), Greatest social deprivation=19 (17.9%). MD Greatest material deprivation=17(21%), Greatest social deprivation=20(24.7%). IP: Greatest material deprivation=275(21.7%), Greatest social deprivation=282(22.3%).*Those using RC less likely to have graduated high school compared to those receiving UC or mixed method delivery (17% V 11% V 14%)* ***(p<0.0001)***.  **URBAN/RURAL:** TM: Urban=2(1.9%), Rural=42(39.6%), Intermediate=59(55.7%). MD: Urban=4(4.9%), Rural=36(44.4%), Intermediate=37(45.7%). IP: Urban=951(75.1%), Rural=164(12.9%), Intermediate=91(7.2%). |
| Lewis 2021 (Australia)  Neurology  Secondary care | F  S  July 2019 – April 2020 | **I:** TH (n=25)  **C:** UC (n=16)  Total (n=41) | **Age:** TH: Median (range)=38(20-73). Mean (SD)=41(16). UC: Median (range)=31(22-81). Mean (SD)=42 (22).  **Gender:** TH: Male=10(40%), Female=15(60%). UC: Male=13(81%), Female=3(19%). *Women significantly more likely to use TH* ***p=0.01***  **DNA:** 3 in intervention group |
| Liu 2021  (Canada)  Geriatric medicine  Secondary care | CS  EHR  17/03/2020 to 13/07/2020 | **I:** V (n=103)  **C:** T (n=227)  Total (n=330) | **AGE:** V: Median (IQR)=84(77 to 87). T: Median (IQR)=83(76 to 88)  **GENDER:** V: Male=44(42.7%). Female=59(57.3%). T: Male=105(46.3%). Female=122(53.7%).  **EDUCATION:** V: Post-secondary=58(56.3%). High school or less=42(40.8%). T: Post-secondary=137(60.4%). High school or less=83(36.6%).  **LANGUAGE:** V: Assessment in English=89(86.4%). T: Assessment in English=198(87.2%.). |
| Lonergan 2020 (USA)  Cancer  Secondary care | RC  HER  01/01/2020 to 13/03/2020; compared to 16/03/2020 to 31/05/2020 | **I:** VC as a result of COVID-19 (n=12,946)  **C:** VP prior to COVID-19 (n=2,284)  Total (n=15,230) | **Age:** VC: Median (IQR)=64.3(54.9 to 71.5). VP: Median (IQR)=63.6(52.8 to 71.8).  **Gender:** VC: Male=6823(52.7%), Female=6123(47.3%). VP: Male=1440(63%), Female=844(37%). **p<0.001.**  **Ethnicity:** VC: Asian=1903(14.7%), Black=531(4.1%), Hispanic/Latino=1450(11.2%), White=7988(61.7%), Other=1075(8.3%). VP: Asian=197(8.6%), Black=78(3.4%), Hispanic/Latino=215(9.4%), White=1606(70.3%), Other=188(8.2%). *Significant increase of Asian, Black and Hispanic/Latino ethnicities having a video consultation during Covid compared to the pre-Covid era* ***p=<0.001.***  **URBAN/RURAL:** VC: Urban=12,014(92.8%), Rural=919(7.1%). VP: Urban=2026(88.7%), Rura= 255(11.2%). Significant i*ncrease in the use of video by patients living in urban areas during Covid compared to the pre-Covid era* ***p=<0.001.*** |
| Menon 2017 (Australia)  Diabetes  Tertiary care | CS  S  May 2016 | **I:** V (n=33  **C:** IP (n=155)  Total (n=188) | **Age:** V: Mean=57.6 +/- 12.6. IP: Mean=54.5 +/- 13.8.  **Gender:** V: Male=13(73%), Female=20(27%). IP: Male=113(39%), Female=42(61%). *Men significantly more likely to have IP consultations* ***(p<0.001),*** *women were significantly more likely to have video* ***(P<0.001).***  **Ethnicity:** V: Aboriginal/Torres Strait=11(33%). IP: Aboriginal/Torres Strait=2(1%). *Higher proportion of indigenous people in the video group compared to the DOS group* ***p<0.001.*** |
| Moo 2020 (USA)  Dementia  Tertiary care | R  S  March 2013 – October 2014 | **I**: V (n=38)  **C:** IP (n=184)  Total (n=222) | **Age:** V: Mean (SD)=79(8.10). IP: Mean (SD)=84(7.03). V *patients significantly more likely to be younger* ***p<0.001.***  **Gender:** V: Male=178(96.7%), Female=6 (3.3%). IP: Male=37(97.4%), Female=1(2.6%).  **Ethnicity:** V: White=28(73.3%). IP: White=141(76.6%). |
| Ohlstein 2020 (USA)  Otolaryngology  Tertiary care | P  D/b  09/03/2020 to 01/05/2020 | **I:** TH (n=146)  **C:** UC (n=379)  Total (n=525) | **Age:** TH: Median=57 years. UC: Median=63 years. *Association between declining telemedicine use and older age* ***p=0.0004.***  **Gender:** TH: Male=59(40%), Female=87(60%). UC: Male=158(42%), Female=221(58%). |
| Poeran 2019  (USA)  Any  Tertiary care | RC  IC  2014 and 2018 | **I:** TM (n=1,018,092)  **C:** UC (n=845,443,517)  Total (n=846,461,609) | **AGE:** TM: <55=858,556(15.0). ≥55=159,536(6.0). UC: <55=576,651,224. ≥55=268,791,293. *OR for older 0.51 (CI: 0.51 to 0.51).* ***p<0.001.***  **GENDER:** TM: Male=344,123(11.0). Female=673,969(13.0). UC: Male=317,436,345. Female=528,007,172.  **INCOME:** TM: <$45k=60489(11.0%). $45k to 60k=339,927(12.0%); >$60k=138,798(17.0%). *OR: 1.38 (CI: 1.38 to 1.39)* ***p<0.001*.** UC: <$45k=55,645,303. $45k to 60k=338,536,472. >$60k=82,325,799.  **URBAN/RURAL:** TM: Urban=879,032(12.0). Rural=103,144(11.0). UC: Urban=706,440,175. Rural=96,957,284. *OR for urban 1.04 (CI: 1.04 to 1.05****) p<0.001.*** |
| Rodriguez 2021 (USA)  Chronic illness  Tertiary care | RC  IC  23/04/2020 to 01/06/2020 | **I:** V (n=37,422)  **C:** T (n=33,466)  Total (n=70,888) | **Age:** V: 18-44=13,366, 45-64=13,030, ≥65=11,029. T: 18-44=6,795, 45-64=10,432, ≥64=16,239.  **Gender:** V: Male=13,453 Female=23,969. T: Male=12,672 Female=20,794.  **Ethnicity:** V: Asian=1,334, Black=1,541, Hispanic=2,072, White=31,749, Other=726. T: Asian=833, Black=1,983, Hispanic=3,463, white=26,565, Other=643.  **EDUCATION:** V: Lowest quartile=11,093, Second quartile=9,594, Third quartile=9,233, Highest quartile=7,502. T: Lowest quartile=7,483, Second quartile=6,900, Third quartile=9,047, Highest quartile 10,036.  **income:** V: Lowest quartile=7,524, Second quartile=9,451, third quartile=9,666, Highest quartile=10,781. T: Lowest quartile=10,573, Second quartile=9,002, Third quartile=6,888, Highest quartile=7,003  **Language:** V: English=36,648, Spanish=415, Other=359. T: English=30,856, Spanish=1,885, Other=725. |
| Rowe 2021  (Australia)  Cardiology  Tertiary care | CS  EHR  17/03/2020 to 12/08/2020 | **I:** T (n=1,188)  **C:** V (n=327)  Total (n=1515) | **AGE:** T: Mean(range) =67(54–76). V: Mean(range) =6146–71). *Patients choosing telephone over a video consultation are more likely to be older* ***p=<0.0001.***  **GENDER:** T: Male=670 (56.4%). Female=518(43.6%). V: Male=213(65.1%). Female=114(34.9%). *Females more likely to choose a telephone compared to a video consultation* ***p=0.005.***  **URBAN/RURAL:** T: Rural=135(11.4%). Urban=1053(88.6%). V: Rural=61(18.7%). Urban=266(81.3%). *Those living in urban areas are more likely to choose telephone consultations* ***p=<0.0001.***  **LANGUAGE:** T: English preferred=1028(86.5%). V: English preferred=294(89.9%)  **INTERPRETER USED:** T: =18(11.3%). V =4(12.1%). |
| Santonicola 2020 (Italy)  Liver transplant  Secondary care | P  D/b  25/02/2020 to 25/04/2020 | **I:** TM (n=79)  **C:** UC (n=74)  Total (n=153) | **Age:** TM: Mean=61.9 +/- 12.1. UC: Mean 65.8 +/- 8.7. *Older patients significantly less likely to use TM* ***p=0.03.***  .  **Gender:** TM: Male=54(68.4%), Female=25(31.6%). UC: Male=51(68.9%), Female=23(31.1%).  **Education:** TM: High school ‘low school degree’=32.5%. UC: High school 'low school degree’=71%. *Those with a low school degree are* s*ignificantly less likely to use TM* ***p=0.001****.* |
| Sellars 2020 (Scotland)  Colorectal disease  Secondary care | P  D/b  March 2019 – February 2020 | **I:** V (n=50)  **C:** IP (n=231)  Total (n=281) | **Did not attend:** Non-attendance rates amongst V patients was 4% compared with 6.1% for IP patients  **Age:** V: Median age: years (range) 68(36-90). <25=0(0%), 26-45=5(10%), 46-65=18(36%), 66-75=17 (34%), 76-85=7(14%), >85=3 (6%). IP: Median age: years (range) 69 (17-90). <25=6(2.6%), 26-45=37 (16%), 46-65=48 (20.8%), 66-75=61 (26.4%), 76-85=68(29.4%), >85=11(4.8%).  **Gender:** V: Male=26 (52%), Female=24(48%). IP: Male=95(41%), Female=136(59%).    **Re-visit rate:** Of the patients seen, two required subsequent review in a face-to-face clinic (4%). |
| Shehan/2021  (USA)  Otolaryngology  Tertiary care | R  EHR  01/10/2019 to 31/10/2019  And  16/03/2020 to 10/04/2020 | **I:** TM in 2020 (n=3,491)  **C:** IP in 2019 (n=4,522)  Total (n=8013) | **age:** TM: Mean=42.68(21.45). IP: Mean=41.58(21.86).  **GENDER:** TM: Male=1481(42.4%). Female=2010(57.6%). IP: Male=1988 (44.0%). Female=2534 (56.0%).  **ETHNICITY:** TM: Asian=133(3.8%), Black=886(25.4%), Hispanic/Latino=324(9.3%), White=996(28.5%), Native American=32(0.9%), Other=46(1.3%), Unknown=1069(30.6). IP: Asian=222(4.9%), Black=1235(27.3%), Hispanic/Latino=303(6.7%), White=1290(28.5%), Native American=37(0.8%), Other=39(0.9%), Unknown=396 (30.9%).  **INCOME:** TM: Median=56,968 SD(20,830). IP: Median=58,134 SD(22,330)  **LANGUAGE:** TM: English=2225(63.7%), Spanish=750(21.5%), Portugese=128(3.7%), Creole=87(2.5%), Cape Verde/Port Creole=82(2.4%), Vietnamese=55(1.6%), Arabic=28(0.8%), Other=129(3.7%). Interpreter used=726 (20.8%). IP: English=2829(62.6%), Spanish=945(20.9%), Portugese=167(3.7%), Creole=167 (3.7%), Cape Verde/Port Creole=108(2.4%), Vietnamese=90(2.0%), Arabic=59(1.3%), Other=154(3.4%). Interpreter=877(19.4%). |
| Stevens/2021  (USA)  Any  Tertiary care | RC  EHR  02/03/2020 to and 10/06/2020 vs. same in 2019 | **I:** TH (n=74,846)  **C:** IP (n=54,998)  Total (n=129,844) | **AGE:** TH: 18-29=5,942(53.4%), 30-39=9,441(48.0%), 40-49=9,208(58.2%), 50-59=13,801(59.4%), 60-69=16,585(59.0%), 70-79=13,058(62.3%), ≥80=6,811(62.6%). IP: 18-29=5,179(46.6%), 30-39=10,234(52.0%), 40-49=6,623(41.8%), 50-59=9,437(40.6%), 60-69=11,535(41.0%), 70-79=7,914(37.7%), ≥80=4,076(37.4). *Older adults less likely to use video technology compared with younger patients* ***P=<0.001.***  **ETHNICITY:** TH: Asian=3,162(55.9%), Black=9,414(61.0%), Hispanic/Latino=3,587(57.5%), White=37,620(59.3%), Other=4,692(55.1%), Unknown=16,381(53.5%). IP: Asian=2,499(44.1%), Black=6,00939.0%), Hispanic/Latino =2,656(42.5%), White=25,777(40.7%), Other=3,817(44.9%), Unknown=14,240(46.5%). |
| Wegerman 2021 (USA)  Liver disease  Secondary care | RC  D/b  01/01/2020 to 29/02/2020 compared with 01/04/2020 to 30/05/2020 | **I:** V (n=958)  **C:** T (n=454)  Total (n=1,412) | **Age:** V: Median (IQR) 58(45 to 67). Telephone: Median (IQR) 63(56 to 70). *Older patients significant more likely to use telephone than video* ***p<0.001.***  **Gender:** V: Male=438, Female=520. T: Male=194, Female=260.  **Ethnicity:** V: Black=142(25.6%), Hispanic/Latino=31(32.3%), White=685(34%), Other=57(31.7%), Not recorded=43(27.9%). T: Black=131(23.6%), Hispanic/Latino=13(13.5%), White=268(13.3%), Other=24(13.3%), Not recorded=18(11.7%). *Black ethnicity associated with significantly higher odds of completion of telephone over a video consultation compared to whites* ***P=<0.001.*** |
| Xiong/2021  (USA)  Orthopaedics  Tertiary care | R  EHR  24/03/2020 to 18/05/2020 and the same period in 2019. | **I:** TM (n=1,760)  **C:** IP (n=9,296)  Total (n=11,056) | **AGE:** TM: Median (range) =57(16-97). IP: Median (range) =59(16-102).  **GENDER:** TM: Male=781(44%), Female=979(56%). IP: Male=4073(44%), Female=5,223(56%). *No difference between gender using RC (p=0.66)*  **ETHNICITY:** TH: Asian=50(3%), Black=107(6%), Hispanic/Latino=26(1%), White=1,460(83%), Other=67(4%), Unknown=50(3%). IP: Asian=316(3%), Black=545(6%), Hispanic/Latino=231(2%), White=7,511(81%), Other=389(4%), Unknown=304(3%). *Hispanic patients significantly less likely to use TM than whites (OR 0.59 [95% CI: 0.39 to 0.91];* ***p = 0.02,*** *as are* *Asian patients (OR 0.73, CI: 0.53 to 0.66,* ***p=0.04****).*  **INCOME**: TH: Median (range) =95k (30.5k to 235.7k). IP: Median (range) =95k (17k to 250k).  **LANGUAGE:** TH: English=1,702(97%), Spanish=272%), Other=10(1%), Unknown=21(1%). IP: English=8,762(94%), Spanish=204(2%), Other=180(2%), Unknown=150(2%). *Patients whose primary language was not English or Spanish less likely to use TM compared to those with English or Spanish as primary language (OR 0.34 [CI 0.18 to 0.65];* ***p = 0.001****).* |
| Yuan/2021  (USA)  Cardiology  Secondary care | CS  EHR  01/04/2019 to 31/12/2019 compared to the same period in 2020 | **I:** 2020 IP (n=74,498), V (n=4,720) and T (n=10,381)  **C:** IP 2019 (n=87,182)  Total (n=176,781) | **AGE:** Mean (SD). IP 2020: =69.04(16.65) *Covid v pre-covid =* ***p<0.001.*** V 2020: =61.09(16.49) *In-person v video =* ***p<0.001.*** T 2020: =68.44(15.99) *In-person v telephone =* ***p<0.001.*** IP 2019: =67.66(17.32).  **GENDER:** IP 2020: Male=41,051(55.1%). Female=33,441(44.9%). *Covid v pre-covid =* ***p=0.01****.* V 2020: Male=2,752(58.3%). Female=1,968(41.7%). *In-person v video* ***= p<0.001****.* T 2020: Male=5,144(49.6%). Female=5,237(50.4%). *In-person v telephone =* ***p<0.001***. IP 2019: Male=48,249(55.3%). Female=38,926(44.6%).  **ETHNICITY:** IP 2020: Asian=4,202(5.6%), Black=5,321(7.1%), Hispanic/Latino=3,482(4.7%), White=54,789(73.5%), Other=3,618(4.9%), Pacific Islander=103(0.1%), American Indian=126(0.2%), Unknown=2,857(3.8%). *Covid v pre-covid* ***= p=<0.001.*** V 2020: Asian=378 (8.0%), Black= 90(8.3%), Hispanic/Latino=255(5.4%), White=3,283(69.6%), Other=235(5.0%), Pacific Islander=7(0.1%), American Indian=7(0.1%), Unknown=165(3.5%). *In-person v video* ***p=0.01.*** T 2020: Asian 810(7.8%), Black=1,073(10.3%), Hispanic/Latino=664(6.4%), White=6,744(65.0%), Other=530(5.1%), Pacific Islander=21(0.2%), American Indian= 9(0.2%), Unknown=520(5.0%). *In-person v telephone =* ***p<0.001.*** IP 2019: Asian=5,973(6.9%), Black=6,366(7.3%), Hispanic/Latino=4,661(5.3%), White=62,264(71.4%), Other=4,408(5.1%), Pacific Islander=122(0.1%), American Indian=180(0.2%), Unknown=3,208(3.7%). |

**Key:** C = cohort, CR = chart review, CS = cross-sectional, D/b = database, DNA = did not attend, EHR = electronic health records, F = feasibility, IC = insurance claims, IP = In-person, MC = matched cohort, MD = mixed delivery, P = prospective, R = retrospective, RC = retrospective cohort, RV = retrospective review, S = survey, SES = socio-economic status, T = Telephone, TH = telehealth, TM = telemedicine, UC = Usual care, V = video, VC = video during COVID-19, VP = video prior to COVID-19, .
